# The comforting companion: using AI to bring loved one's voices to newborns, infants, and unconscious patients in ICU

**DOI:** 10.1186/s13054-023-04418-5

**Published:** 2023-04-06

**Authors:** Hongkun Zhou, Xiaojun Wu, Linghua Yu

**Affiliations:** 1 Hepatopancreatobiliary Surgery Department, The Affiliated Hospital of Jiaxing University, 1882 Central-South Road, Jiaxing, 314001 Zhejiang Province People’s Republic of China; 2 Gastroenterology and Hepatology Department, Institute of Liver Diseases, The Affiliated Hospital of Jiaxing University, 1882 Central-South Road, Jiaxing, 314001 Zhejiang Province People’s Republic of China

Emotional support from loved ones is of the utmost importance for a patient's recovery in the challenging environment of the intensive care unit (ICU). This environment can be particularly difficult for vulnerable groups, such as newborns, infants, and minimally conscious patients. Numerous studies have proved that the presence of family members can significantly enhance a patient's physical and mental well-being, by reducing anxiety and stress [1, 2]. Additionally, emotional support has been identified as crucial for arousal in comatose patients [3–5]. However, providing reassurance can be challenging, especially when patients cannot communicate effectively. The absence of family members due to hospital regulations exacerbates the issue.

To address this issue, we introduce the reassurance voice mimic system which utilizes artificial intelligence (AI) technology to generate personalized soothing voice messages for each patient based on their medical condition and psychological needs. These messages can be delivered via various terminal devices, such as smart speakers and interactive toys. By providing personalized emotional support, the reassurance voice mimic system has the potential to improve the mental well-being of patients and enhance the quality of care provided by healthcare professionals.

In the intensive care unit, providing personalized care that considers the unique needs of each patient is crucial for successful treatment and recovery. To this end, we developed a prototype system that generates soothing voice messages for newborns, infants, and comatose patients by mimicking the voices of their family members (Fig. 1). The generation of individualized reassurance messages involves a complex process, which includes generating reassurance texts, reviewing and approval of the text, voice imitation, and audio message synthesis. To achieve this, the reassurance voice mimic system uses a pipeline architecture that combines various AI technologies, such as natural language processing, voice cloning, and audio synthesis. The system utilizes a language model to analyze patients' medical records, such as their medical history, progress notes, and treatment plans. By extracting relevant information about the patients, the system will then generate soothing messages that are tailored to their specific needs. These reassuring messages include phrases such as “Honey, honey, I'm your mother”, “Don't be afraid now, I will be by your side.”, “I'm so proud of you for staying strong.”, “Every day you are making progress, keep it up.”, etc. The reassurance texts are then reviewed by a healthcare provider to ensure they are appropriate and safe for the patient. The healthcare provider can either approve the text as is or revise it as needed to ensure that it does not conflict with any medical instructions or treatment plans. By providing a one-minute sound recording of the family member, a voice-cloning model will generate a voice message from the reassurance text that imitates the voice of a loved one. Next, the system will synthesize the audio message by adding personalized background music that will make the patient feel calm or joyful. Finally, the reassurance audio message can be delivered to the patient through a variety of terminal devices, such as smart speakers for adults or interactive toys for infants. Patients can interact with the system using voice commands, or other interfaces, depending on their physical and cognitive abilities.

**Fig. 1:**
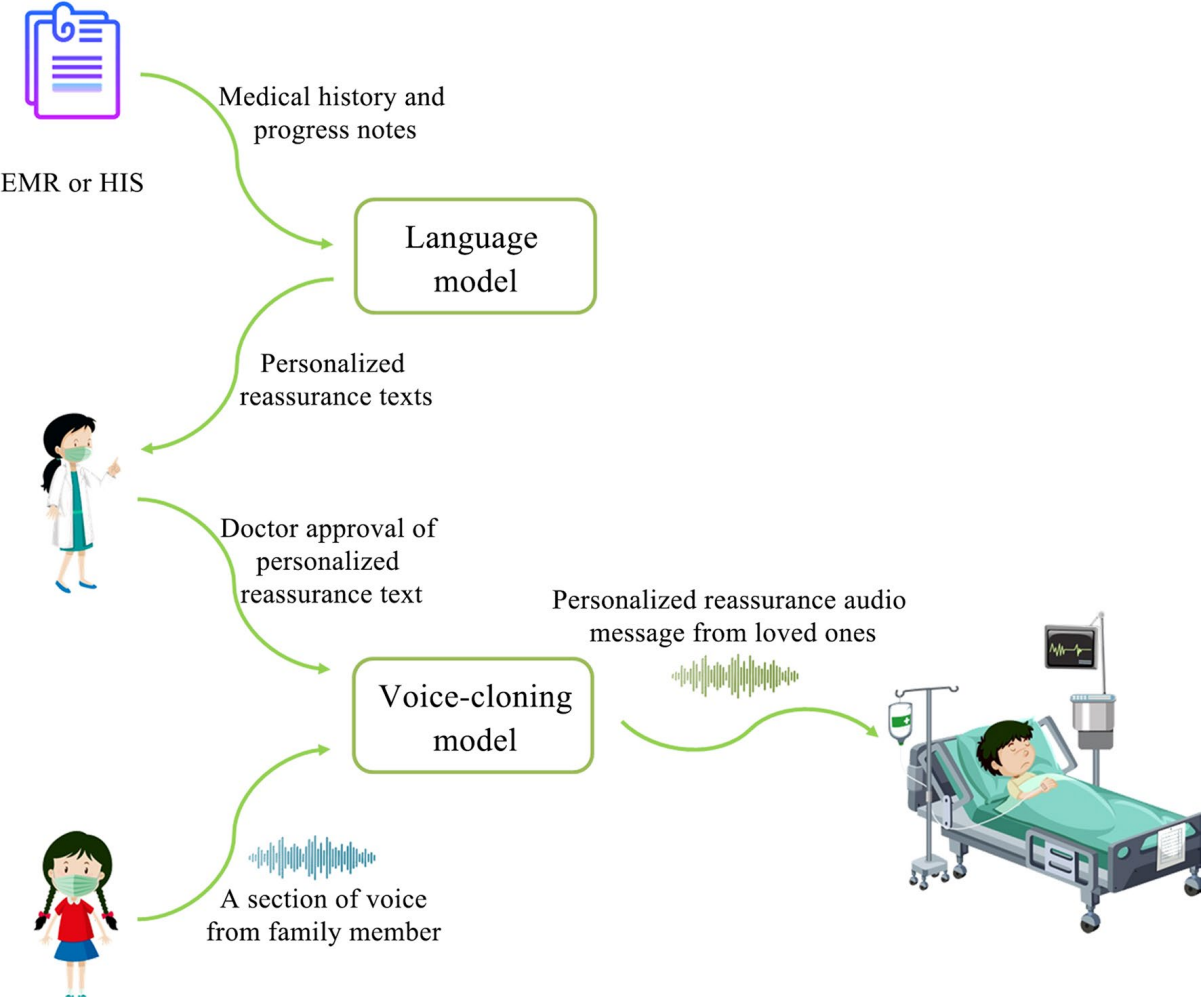
The schematic of the AI-assisted reassurance system

A typical scenario for using our system is as follows: When it is necessary to communicate with an infant or minimally conscious patient, the nursing staff will initiate the system by pressing a button on the terminal device. The bedside device will then start to comfort the patient in the voice of the patient's loved one. The AI system will make adjustments to the patient's reassurance plan based on their progress notes at any given time.

One of the key benefits of our system is its ability to deliver reassurance messages that are specifically tailored to each patient's emotional needs. This personalized approach helps establish a stronger sense of emotional connection between patients and their loved ones, even in situations where physical presence is not possible due to ICU regulations. The system's employment of voice-cloning technology to replicate the familiar and soothing voice of loved ones is especially comforting and reassuring for infants and minimally conscious patients. By serving as a supportive measure for patients, the reassurance voice mimic system can effectively complement the care provided by healthcare providers in the ICU, potentially reducing their workload and stress levels, and enabling them to focus on other critical aspects of patient care. Moreover, the system has the potential to positively impact patient outcomes. Extensive research has suggested that emotional stress and anxiety can adversely affect patient outcomes while providing emotional support can help diminish the risk of complications and hasten recovery times [6, 7].

In summary, the reassurance voice mimic system offers a promising solution for delivering individualized emotional support to infants and minimally conscious patients in the ICU. Its use of artificial intelligence technology to deliver personalized messages can improve emotional well-being, alleviate stress and anxiety, and enhance the quality of care provided by healthcare providers. Although certain challenges and considerations need to be addressed, the potential benefits of this system make it a valuable and promising tool for critical care settings.

## Data Availability

Not applicable.
